# Acute Cholecystitis Caused by Ceftriaxone Stones in an Adult

**DOI:** 10.1155/2009/132452

**Published:** 2009-04-26

**Authors:** Christian D. Becker, Robert A. Fischer

**Affiliations:** ^1^The Mount Sinai Medical Center, New York, NY 10029, USA; ^2^Albert Einstein Medical Center, Philadelphia, PA 19141, USA

## Abstract

Acute cholecystitis is a major health problem. There are multiple etiologies to be considered and early recognition of the condition is important to optimize management and outcome. We report the first case in the medical literature of symptomatic acute cholecystitis triggered by ceftriaxone-associated gallbladder sludge formation and, importantly, solid ceftriaxone gallstone formation in an adult patient with underlying mineral and pigment cholecystolithiasis, necessitating cholecystectomy. This case serves as a reminder for physicians to keep this uncommon cause of cholecystolithiasis and cholecystitis in mind in patients who receive prolonged ceftriaxone therapy. These patients should be cautioned to promptly report to their physicians any signs or symptoms of cholecystitis in order to ensure timely and appropriate evaluation.

## 1. Background

Acute cholecystitis is a major health problem. There are multiple etiologies to be considered and early recognition of the condition is important to optimize management and outcome.

## 2. Case Report

A-68-year-old African-American male was admitted for 2 days of worsening right upper quadrant abdominal pain. The pain was constant, noncolicky, nonradiating, 6–8/10 in intensity, accompanied by nausea and vomiting with attempted food intake. No fevers or chills were present.

9 weeks prior to admission the patient underwent craniotomy for drainage of multiple brain abscesses. Cultures were negative. Long-term empiric therapy with high doses of ceftriaxone (2 gm i.v. q12 hours), caspofungin (50 mg i.v. q24 hours), metronidazole (500 mg p.o. q8 hours), and corticosteroids was initiated at that time.

Physical examination revealed marked right upper quadrant tenderness and a positive Murphy's sign. Laboratory data showed a normal white blood cell count of 5400/mm^3^, hemoglobin of 10.3 g/dL, normal blood chemistry values except a blood glucose of 157 mg/dL. The total bilirubin was normal at 0.2 mg/dL, the alkaline phosphatase was slightly elevated at 177 IU/L. The transaminases were normal (AST 21 IU/L, ALT 14 IU/L).

A CT scan revealed gallbladder wall thickening, pericholecystic fluid, and multiple gallstones in the gallbladder.

**Figure 1:**
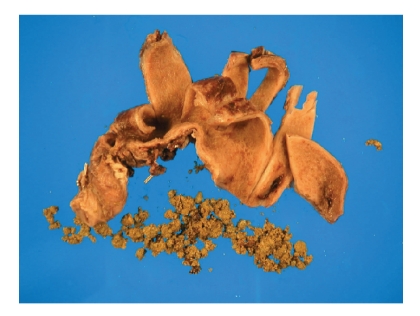
Surgical specimen (gallbladder, with resection clips) showing a large number of greenish-brownish gallstones of various sizes and “crumbly” consistency.

The patient underwent cholecystectomy on the day of admission. The gallbladder contained numerous green-yellow gallstones of various sizes (see Figure 1), which were soft and easily compressible.

The patient experienced an uncomplicated postoperative course.

The prolonged treatment with high doses of ceftriaxone and peculiar consistency of the gallstones resulted in the clinical suspicion of ceftriaxone-associated gallstones. Therefore, a gallstone sample was analyzed by high-pressure liquid chromatography [1]. The sample contained 70.4% minerals, 7.5% pigments, and 0.1% cholesterol as well as significant amounts of ceftriaxone calcium salt (mean of 22%, with 3 separate measurements of 20.97%, 23.43%, and 21.59%).

## 3. Discussion

Ceftriaxone is 10–40% hepatically eliminated [2] and is known to induce reversible precipitates of ceftriaxone calcium in the gallbladder [3]. Ultrasonographically, these precipitates resemble gallbladder sludge, but the ultrasound-morphologic similarity to gallstones as well as the rapid disappearance after discontinuation of ceftriaxone has resulted in the term “ceftriaxone pseudolithiasis.”

The phenomena of ceftriaxone-associated sludge formation and ceftriaxone-associated cholelithiasis are well documented in the pediatric literature and have resulted in unnecessary cholecystectomies in pediatric patients. The incidence of ceftriaxone sludge formation in patients treated with ceftriaxone ranges from 25% to 46%, but only a minority of these patients become symptomatic [4].

A Medline search with the terms (“ceftriaxone” AND (“acute cholecystitis” OR “cholecystolithiasis”)) from 1980 to 2008 revealed one report of ceftriaxone-associated cholecystolithiasis leading to acute gallstone pancreatitis in a 42-year-old male [5], but no reports of ceftriaxone-associated cholecystolithiasis with acute cholecystitis in adults.

However, there are several reports of coexisting cholecystolithiasis in patients with ceftriaxone-associated gallbladder sludge formation. There are three mechanisms by which a patient receiving ceftriaxone can develop acute cholecystitis: ceftriaxone-associated sludge can trigger existing gallstones to become symptomatic, ceftriaxone pseudolithiasis can transform into ceftriaxone gallstones, or the patient can become symptomatic from preexisting cholecystolithiasis unrelated to ceftriaxone therapy.

Because of the unusual appearance and consistency of the stones in our patient and the analysis showing that ceftriaxone was incorporated into the stones in significant amounts, we believe that ceftriaxone therapy was responsible for the development of acute cholecystitis in our patient.

## 4. Conclusion

We report the first case in the medical literature of symptomatic acute cholecystitis triggered by ceftriaxone-associated gallbladder sludge formation and, importantly, solid ceftriaxone gallstone formation in an adult patient with underlying mineral and pigment cholecystolithiasis, necessitating cholecystectomy.

This case serves as a reminder for physicians to keep this uncommon cause of cholecystolithiasis and cholecystitis in mind in patients who receive prolonged ceftriaxone therapy. These patients should be cautioned to promptly report to their physicians any signs or symptoms of cholecystitis in order to ensure timely and appropriate evaluation.
